# DENV2 and ZIKV modulate the feeding behavior of *Aedes aegypti* by altering the tyrosine-dopamine pathway

**DOI:** 10.1128/mbio.03968-24

**Published:** 2025-04-29

**Authors:** Dongmin Gao, Ruixu Jiang, Zhaoyang Wang, Jichen Niu, Gang Wang, Yicheng Wang, Yan Liang, Yibin Zhu, Gong Cheng

**Affiliations:** 1 New Cornerstone Science Laboratory, School of Basic Medical Sciences, Tsinghua University-Peking University Joint Center for Life Sciences, Tsinghua University, Beijing, China; 2 Institute of Infectious Diseases, Shenzhen Bay Laboratory 551667, Shenzhen, China; 3 Institute of Pathogenic Organisms, Shenzhen Center for Disease Control and Prevention 568734, Shenzhen, China; Washington University in St. Louis School of Medicine, St. Louis, Missouri, USA

**Keywords:** flavivirus, feeding behavior, circadian rhythm, virus transmission

## Abstract

Flaviviruses are known for their neurotropic properties and their long-lasting neurological effects. As mosquito-borne viruses, they can persistently infect the central nervous system of mosquitoes without causing symptoms, yet they can alter the physiology and behavior of these insects. However, the molecular mechanisms by which flaviviruses directly influence mosquito feeding behavior remain largely unexplored. Here, we show that dengue virus serotype 2 (DENV2) and Zika virus (ZIKV) elevate mosquito locomotor activity and blood-feeding propensity throughout the day. Untargeted metabolomics identified increased N-acetyl-L-tyrosine levels in virus-infected mosquito heads, which enhanced locomotor activity and blood-feeding efficiency when injected into *Aedes aegypti*. Virus infection disrupted the circadian rhythm of *tyrosine hydroxylase* (*Aath*), a key dopamine synthesis gene, maintaining its consistent elevation of expression throughout the day. Furthermore, DENV2 and ZIKV perturbed the molecular circadian core oscillator in *Ae. aegypti*. Additionally, mosquitoes injected with L-3,4-dihydroxyphenylalanine exhibited higher locomotor activity and blood-sucking rates. In contrast, knockdown of *Aath* reduced blood feeding and decreased infection rates in mice bitten by virus-infected mosquitoes. Collectively, our findings elucidate the molecular mechanisms by which DENV2 and ZIKV modulate the physiology and feeding behavior of *Ae. aegypti* vectors, thereby facilitating the transmission of these viruses.

**IMPORTANCE:**

This study sheds light on how DENV2 and ZIKV affect the feeding behavior of mosquitoes. We discovered the molecular mechanisms that lead to increased movement and blood feeding in mosquitoes by altering neurotransmitter levels and disrupting their internal biological clocks. These findings reveal how these viruses enhance their own transmission by making mosquitoes more active. This research could help in developing strategies to target these processes, ultimately aiding efforts to control the spread of dengue and Zika viruses and reducing the risk of outbreaks.

## INTRODUCTION

Mosquitoes are primary vectors of numerous pathogens causing harmful diseases in humans and animal hosts, including yellow fever, dengue fever, malaria, Chikungunya fever, and Zika fever. These diseases collectively result in hundreds of millions of infections and millions of deaths annually across more than 125 countries, posing a serious global public health threat (1). The feeding behavior of viruliferous mosquitoes is a critical component in the transmission of insect-borne pathogens, and understanding this behavior is key to controlling arbovirus outbreaks (2). However, the mechanisms by which mosquito-borne viruses manipulate the feeding behavior of their vectors to enhance transmission remain poorly understood.

Flavivirus transmission occurs during the mosquito’s blood-feeding process. Initially, the virus is excreted through the mosquito’s saliva into the host’s skin. The virus then infects skin cells and is transported by myeloid cells to lymph nodes, ultimately triggering systemic infection (3). Blood feeding is thus an essential prerequisite for systemic infection and subsequent viral spread. Recent studies have shown that *Aedes* mosquitoes exhibit increased host-seeking and feeding behavior when exposed to dengue virus serotype 2 (DENV2)- or Zika virus (ZIKV)-infected hosts, suggesting that infected animals emit cues that enhance mosquito attraction (4). Additionally, flaviviruses directly modulate mosquito biting behavior to facilitate transmission. For instance, DENV1 has been shown to alter locomotion and odor-mediated host-seeking behavior in *Aedes aegypti*, while DENV2 increases host attraction and the frequency of infectious probes (5–7). However, some studies, such as those on *La Crosse* virus (LACV), found no significant effects on mosquito blood-feeding behavior or associated dopamine (DA) levels (8).

DA, a critical catecholamine neurotransmitter, plays diverse roles in the physiology of both vertebrates and invertebrates, including memory, learning, courtship, feeding, and sleep regulation (9–13). In insects, dopamine signaling regulates feeding behavior, salivary function, and host-seeking activity. For example, dopamine mediates the food wanting system in honeybees (14), memory suppression in *Drosophila melanogaster* (15, 16), and saliva secretion in *Aedes aegypti* (17). Tyrosine, a precursor of dopamine, is catalyzed by tyrosine hydroxylase (TH), the rate-limiting enzyme for dopamine synthesis, into L-3,4-dihydroxyphenylalanine (L-DOPA), which is subsequently converted into dopamine by dopa decarboxylase (DDC) (13). Notably, dopamine synthesis in mammals exhibits circadian rhythm regulation, suggesting that similar regulatory mechanisms may exist in mosquitoes (18, 19). Mosquitoes express 24 h rhythms in behavior and physiology. Many of these rhythms are driven by an endogenous circadian clock (20).

The molecular machinery governing circadian rhythms has been extensively studied in *Drosophila melanogaster*. Key clock components include CLOCK (CLK) and CYCLE (CYC), which form a heterodimer and promote transcription of *period* (*per*), *timeless* (*tim*), *clockwork orange*, and *PAR domain protein 1*ε (*Pdp1*) genes. The PER and TIM proteins subsequently accumulate and translocate to the nucleus, repressing CLK-CYC activity, thereby completing a negative feedback loop that cycles every 24 h (21–24).

In this study, we demonstrate that infection with DENV2 and ZIKV disrupts the circadian rhythm and tyrosine-dopamine pathway in *Aedes aegypti*, leading to enhanced locomotor activity and increased blood-feeding propensity. Untargeted metabolomic analysis revealed that infection with DENV2 and ZIKV induces the accumulation of N-acetyl-L-tyrosine in the mosquito head, which further stimulates dopamine production. Our findings suggest that the circadian rhythm of the dopamine synthesis gene, *tyrosine hydroxylase* (*Aath*), is disrupted by infection with DENV2 and ZIKV. Knocking down the *Aath* gene in infected mosquitoes significantly reduced viral transmission efficiency to the host. Collectively, these results provide new insights into how DENV2 and ZIKV manipulate mosquito physiology and feeding behavior to promote transmission.

## RESULTS

### DENV2 and ZIKV infections in *Aedes aegypti* brain enhance locomotor activity and blood-feeding propensity

**Fig 1:**
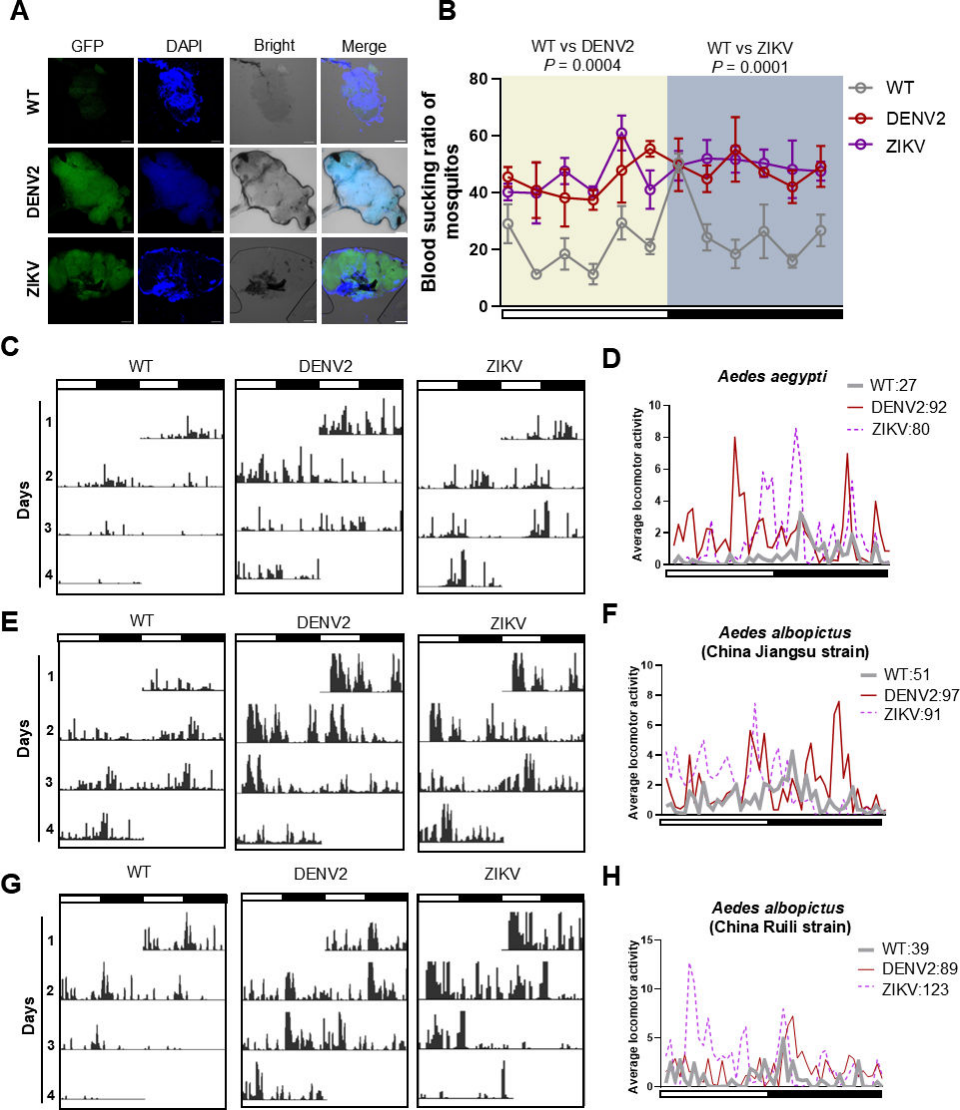
Infection with DENV2 and ZIKV in *Aedes aegypti* brain enhances locomotor activity and blood-feeding propensity. (**A**) Alexa Fluor 488 fluorescence of *A. aegypti* brains at 7 days after DENV2 and ZIKV injection. The tissues were stained with 4′,6-diamidino-2-phenylindole, dihydrochloride (DAPI) to indicate nuclei. Scale bars, 100 µm. (**B**) The blood-feeding ratio of uninfected, DENV2- and ZIKV-infected mosquitoes after biting AG6 mice in 30 min, tested at 2 h intervals throughout the day. White and black bars indicate light and dark phases, respectively. Two-way analysis of variance followed by Tukey’s test was performed to investigate the main effects of DNEV2 and ZIKV infection on mosquitoes’ blood-feeding ratio. Differences were considered significant at *P* < 0.05. (**C, E, and G**) The locomotor activity of uninfected, DENV2- and ZIKV-infected *Aedes aegypti* (**C**), *Aedes albopictus* (China Jiangsu strain) (**E**), and *Aedes albopictus* (China Ruili strain) (**G**) in light/dark cycles. White and black bars indicate light and dark phases, respectively. (**D, F, and H**) Comparison of the circadian locomotor activity of non-infected and infected mosquitos in LD cycles. The lines represent the distribution of activity counts (y axis) every 30min of indicated mosquitoes measured in panels C, E, and G through 24 h, averaged over 4 LD days.

Flaviviruses, as mosquito-borne pathogens, can persistently replicate within mosquitoes, infecting various tissues, including the brain, which houses the central nervous system (CNS) in *Aedes aegypti* (25). We confirmed this by immunofluorescence assays, where Alexa Fluor 488 fluorescence was observed in the dissected brains of viruliferous mosquitoes, indicating the presence of DENV2 and ZIKV envelope proteins (Fig. 1A). Previous studies have shown that ZIKV infection increases neuronal spiking activity and synaptic connections (26), and it is well established that the CNS regulates animal behavior. Therefore, it is reasonable to hypothesize that DENV2 and ZIKV infections in the mosquito brain may alter mosquito behavior.

The blood-feeding behavior of *Ae. aegypti* is critical for viral transmission. To assess the impact of infection on blood-feeding propensity, we measured the blood-sucking rate of female mosquitoes every 2 h throughout the day. Uninfected mosquitoes exhibited the highest feeding rate in the evening, followed by dawn, with lower rates during other times of the day. In contrast, DENV2- and ZIKV-infected mosquitoes showed consistently higher blood-feeding rates throughout the day (Fig. 1B). Increased blood-feeding propensity in infected mosquitoes likely facilitates viral transmission to a larger number of hosts.

In addition, we monitored the locomotor activity of mosquitoes using the Drosophila Activity Monitor 2 (DAM2) system. Both DENV2- and ZIKV-infected mosquitoes demonstrated significantly increased activity compared to uninfected controls (Fig. 1C and D). These assays were conducted using the *Ae. aegypti* Rockefeller strain, while *Aedes albopictus*, another primary vector for DENV and ZIKV, was also included in the study. Two field strains of *Ae. albopictus*, one from Ruili in Yunnan province (China Ruili strain) and the other from Jiangsu province (China Jiangsu strain), were tested. The results were consistent with those observed in *Ae. aegypti*, with both DENV2- and ZIKV-infected *Ae. albopictus* showing increased activity (Fig. 1E through H).

In summary, our findings demonstrate that infection with DENV2 and ZIKV in the brain of *Aedes* mosquitoes increases both their blood-feeding propensity and locomotor activity, which may enhance viral transmission.

### Untargeted metabolomic identifies metabolites altered in the heads of mosquitoes infected by DENV2 and ZIKV

To investigate how DENV2 and ZIKV affect mosquito behavior, we conducted an untargeted metabolomics analysis to identify changes in the metabolite composition of mosquito brains. Quality control (QC) analysis revealed that the Pearson correlation between positive and negative QC samples was greater than 0.99 (Fig. S1), indicating a high degree of consistency between the samples. Principal component analysis (PCA), shown in Fig. S2, demonstrated that the data from all samples were within a 95% CI. The three groups were clearly separated along the first principal component, with each group closely clustered, suggesting good repeatability of the samples. Additionally, the groups were distinctly separated from one another, indicating substantial differences between the groups.

**Fig 2:**
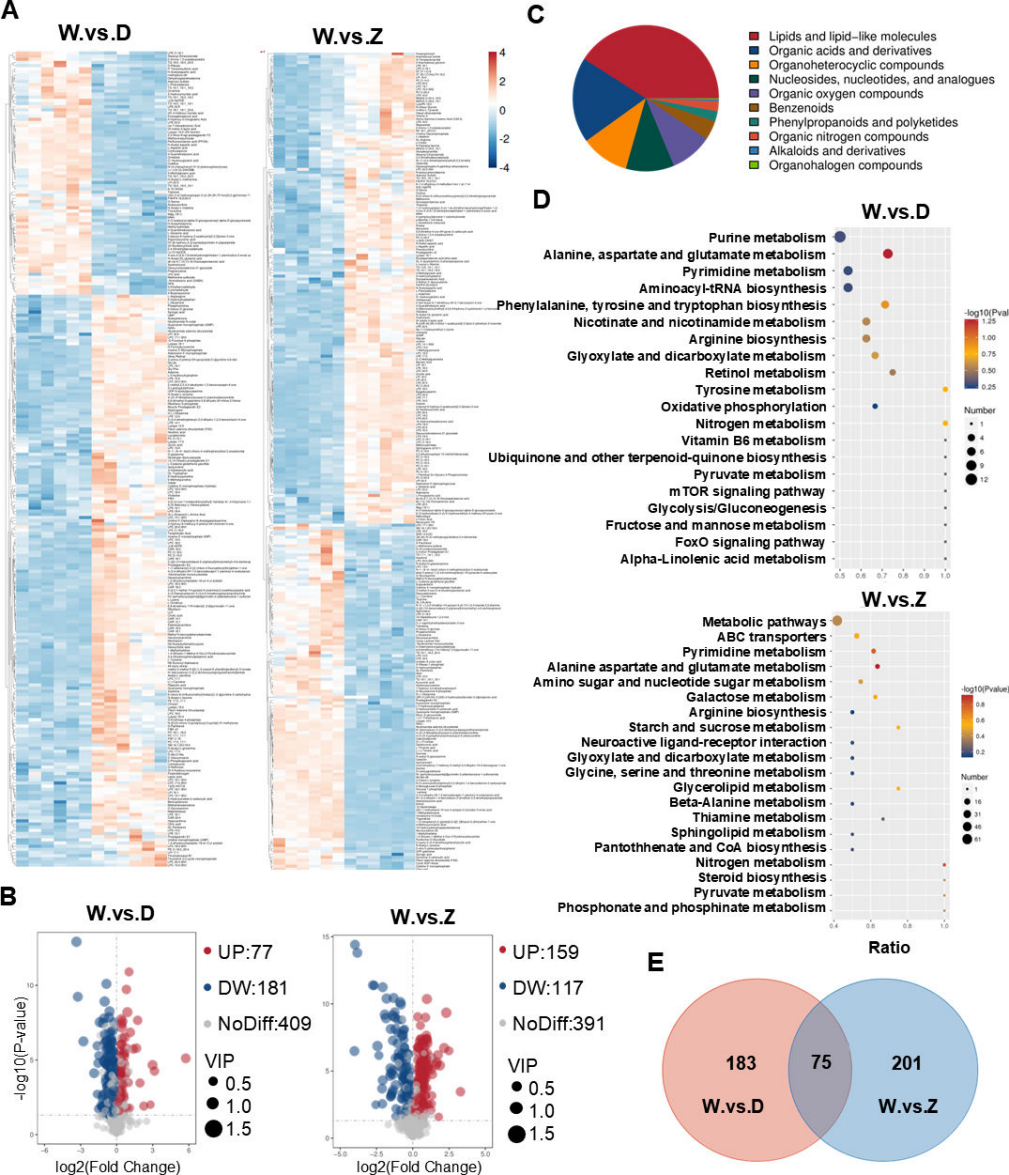
Untargeted metabolomic identifies metabolites altered in the heads of *Ae. aegypti* infected by DENV2 and ZIKV. (**A**) Heatmap of the differences in metabolic expression patterns between wild-type (WT) mosquito and virus-infected mosquito. The left panel shows WT vs. DENV2-infected mosquitoes (W.vs.D), while the right panel shows WT vs. ZIKV-infected mosquitoes (W.vs.Z). (**B**) The volcano plot represents the overall distribution of differential metabolites. The horizontal coordinate indicates the differential fold change of metabolites in different conditions, and the vertical coordinate indicates the *P* value. Each point in the volcano plot represents a metabolite. Significantly upregulated metabolites are indicated by the red dots, and significantly downregulated metabolites are indicated by the blue dots. The size of the dots represents the variable importance in projection (VIP) value. (**C**) The chemical classification of the identified metabolite. Different areas reflect the proportion of the specific metabolite among all metabolites. (**D**) KEGG analysis of differential metabolites in different groups. Larger values of the horizontal coordinates indicate higher enrichment of differential metabolites in the pathway. The color of the dots represents the *P* value, and the size of the dots represents the number of differential metabolites in the corresponding pathway. (**E**) Venn diagram showing differential metabolites between the combination of W.vs.D and W.vs.Z.

A total of 411 metabolites in positive ion mode and 256 metabolites in negative ion mode were identified across the 18 samples. The results of the Kyoto Encyclopedia of Genes and Genomes (KEGG) functional and taxonomic annotations for the identified metabolites are presented in Fig. S3. Heatmap (Fig. 2A) and volcano plots (Fig. 2B) illustrate the up- and downregulation of metabolites in DENV2- and ZIKV-infected mosquitoes compared to uninfected mosquitoes. A detailed list of all identified metabolites is available in Data S1. Chemical classification of the metabolites revealed that 40.98% were lipids and lipid-like molecules, followed by organic acids and derivatives (18.9%). Organoheterocyclic compounds and nucleosides, nucleotides, and analogues each constituted 10.69%, while organic oxygen compounds accounted for 9.35%. Benzenoids represented 4.23%, with other compound classes accounting for approximately 5% of the total (Fig. 2C).

Differential metabolites between wild-type and DENV2 or ZIKV-infected mosquitoes were identified through KEGG pathway enrichment analysis, with the top 20 enriched pathways shown in Fig. 2D. A Venn diagram (Fig. 2E) illustrates the overlap and unique metabolites among the three combinations of differential metabolites. Ultimately, we identified a total of 75 metabolites that were up- or downregulated in both DENV2 and ZIKV-infected mosquitoes (Table S1).

In conclusion, through untargeted metabolomics, we analyzed the differences in head metabolites between DENV2- or ZIKV-infected mosquitoes and uninfected mosquitoes, revealing that both DENV2 and ZIKV induce alterations in mosquito metabolites.

### N-acetyl-L-tyrosine increases mosquito blood-feeding propensity and locomotor activity

In the untargeted metabolomics analysis, we focused on metabolites that were upregulated during DENV2 and ZIKV infections. Notably, 5-hydroxytryptophan, panthenol, and glutamine were identified in various forms among the upregulated metabolites. Since lipid molecules primarily serve as structural components of cells and cannot be overexpressed in mosquitoes via microinjection, we did not examine the effects of increased lipids and lipid-like molecules. Instead, we microinjected the remaining identified metabolites into the thorax of mosquitoes and assessed their impact on mosquito blood-feeding behavior.

**Fig 3:**
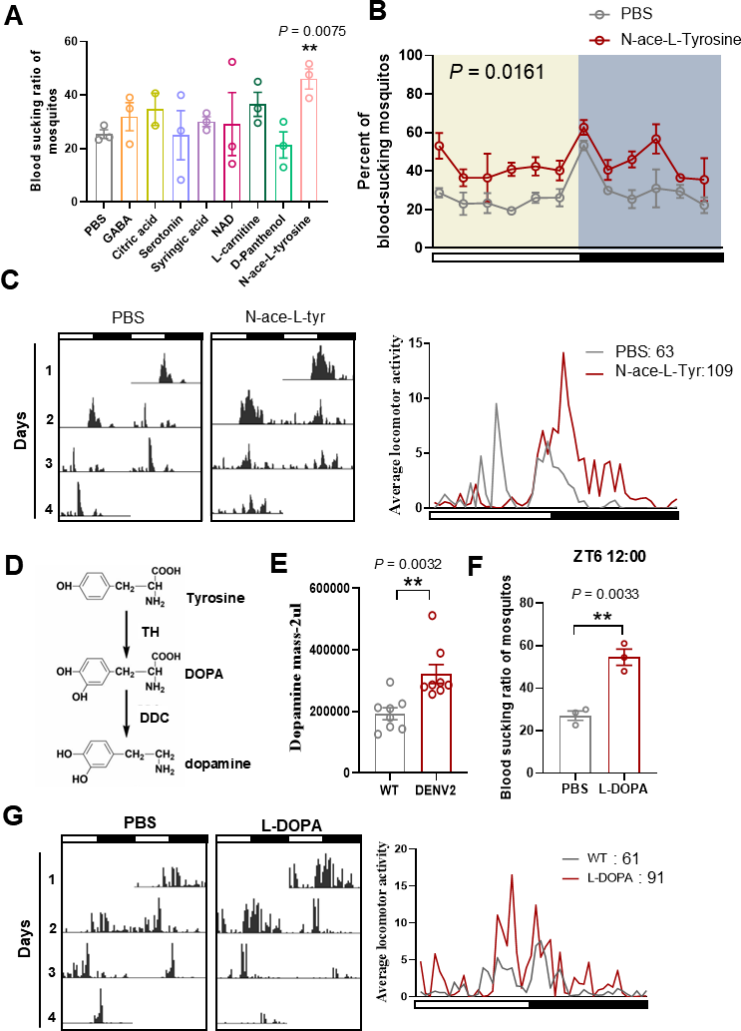
N-acetyl-L-tyrosine increases mosquito blood-feeding propensity and locomotor activity. (**A**) The blood-feeding ratio of mosquitoes injected by partial virus-induced metabolites. The error bar indicates the SEM. **P < 0.01 (two-sided *t*-test). (**B**) The blood-feeding ratio in 24 h of mosquitoes injected by N-acetyl-L-tyrosine. The error bar indicates the SEM. Two-way analysis of variance followed by Tukey’s test was performed to investigate the main effects of N-ace-L-tyrosine on mosquitoes’ blood-sucking ratio. Differences were considered significant at *P* < 0.05. (**C**) The left panel shows the locomotor activity of mosquitoes injected by N-acetyl-L-tyrosine in light/dark (LD) cycles. White and black bars indicate light and dark phases, respectively. PBS-injected mosquitoes served as a negative control. The right panel shows the average locomotor activity measured by the left panel. The numbers represent the total activity counts of mosquitoes over 24 h. (**D**) Schematic diagram of the dopamine synthesis pathway. (**E**) Dopamine levels in the head of uninfected and DENV2-infected mosquitoes. The error bar indicates the SEM. **P < 0.01 (two-sided *t*-test). (**F**) The blood-feeding ratio of mosquitoes injected by L-DOPA at ZT6, with PBS-injected mosquitoes as negative control. The error bar indicates the SEM. **P < 0.01 (two-sided *t*-test). (**G**) The left panel shows the locomotor activity of mosquitoes injected by L-DOPA in LD cycles. White and black bars indicate light and dark phases, respectively. PBS-injected mosquitoes served as a negative control. The right panel shows the average locomotor activity measured by the left panel. The numbers represent the total activity counts of mosquitoes over 24 h. DDC, dopa decarboxylase; TH, tyrosine hydroxylas*e;* ZT, Zeitgeber time.

Our data indicated that N-acetyl-L-tyrosine, which was upregulated in the brains of both DENV2- and ZIKV-infected mosquitoes (Fig. S4), enhanced the blood-feeding propensity (Fig. 3A). Interestingly, mosquitoes injected with N-acetyl-L-tyrosine exhibited a marked increase in locomotor activity (Fig. 3B). This suggests a potential correlation between increased activity and enhanced blood-feeding efficiency. As expected, the blood-feeding ratio of N-acetyl-L-tyrosine-injected mosquitoes was significantly higher than that of phosphate buffer saline (PBS)-injected mosquitoes at various time points over 24 h (Fig. 3C).

L-tyrosine, a precursor in dopamine synthesis, plays roles in regulating mood and protecting the nervous system (27). In this pathway, tyrosine is first converted to L-DOPA by TH and then further converted into DA by DDC (Fig. 3D). Since virus infection induced an increase in tyrosine levels, we hypothesized that the virus may also impact the downstream production of dopamine and alter its levels. High-performance liquid chromatography (HPLC) analysis revealed that dopamine levels in the head tissues of DENV2-infected *Aedes aegypti* were approximately 1.7-fold higher than those in uninfected mosquitoes (Fig. 3E).

To investigate the role of dopamine in mosquito blood-feeding behavior, we injected mosquitoes with 0.01 mg/mL L-DOPA, which resulted in a twofold increase in dopamine levels (Fig. S5). This injection significantly enhanced the blood-feeding ratio at ZT8 and increased the average locomotor activity of the mosquitoes (Fig. 3F and G). These findings suggest that dopamine plays a positive role in enhancing the blood-feeding propensity of mosquitoes.

### Inhibition of the circadian rhythm by DENV2 and ZIKV impacts *Aath* expression in mosquitoes

**Fig 4:**
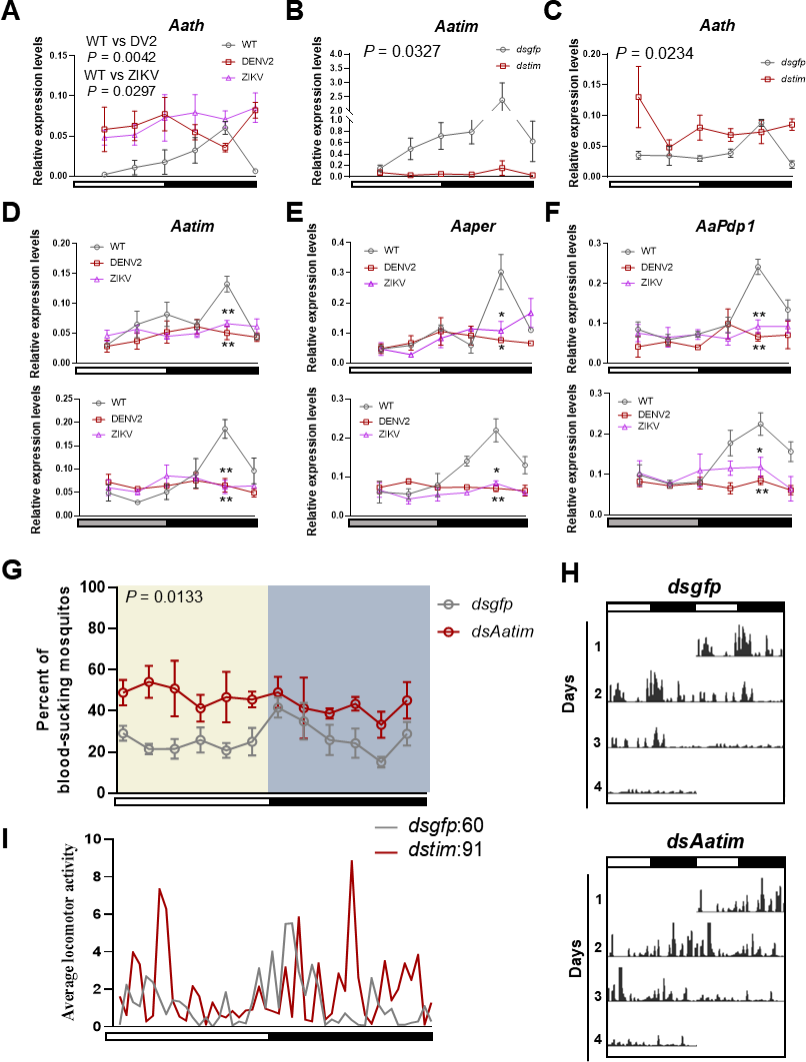
Inhibition of circadian rhythm by DENV2 and ZIKV impacts *Aath* expression in mosquitoes. (**A**) Reverse transcription-quantitative PCR (RT-qPCR) analyzing the relative levels of *Aath* mRNA in the head of uninfected, DENV2-, and ZIKV-infected *Aedes aegypti* under LD cycles. The *RPS17* gene served as an endogenous control. The error bar indicates the SEM. Two-way analysis of variance (ANOVA) followed by Tukey’s test was performed to investigate the main effects of N-ace-L-tyrosine on mosquitoes’ blood-sucking ratio. Differences were considered significant at *P* < 0.05. (**B and C**) RT-qPCR analyzing the relative levels of *Aatim* (**B**) or *Aath* (**C**) mRNA in *dsgfp*- or *dsAath*-injected mosquitoes’ heads under LD cycles. The *Aedes aegypti RPS17* gene served as an endogenous control. The error bar indicates the SEM. Two-way ANOVA followed by Tukey’s test was performed to investigate the main effects of *dstim* on gene expression levels in mosquitoes. Differences were considered significant at *P* < 0.05. (**D through F**) RT-qPCR analyzing the relative levels of *Aatim* (**D**), *Aaper* (**E**), and *AaPdp1* (**F**) mRNA in the head of uninfected, DENV2-, and ZIKV-infected *Aedes aegypti* under LD cycles. The *RPS17* gene served as an endogenous control. The top panel is under LD cycles, and the panel below is under constant dark cycles. The error bar indicates the SEM. *P < 0.05, **P < 0.01 (two-sided t test). (**G**) The blood-feeding ratio in 24 h of *dsgfp*- or *dsAatim*-injected mosquitoes. White and black bars indicate light and dark phases, respectively. The error bar indicates the SEM. Differences were considered significant at *P* < 0.05. (**H**) The locomotor activity of *dsgfp*- or *dsAatim*-injected mosquitoes under LD cycles. The column represents sums of 16 mosquitoes. White and black bars indicate light and dark phases, respectively. (**I**) The average locomotor activity in panel H. The numbers represent the total activity counts of mosquitoes over 24 h.

Tyrosine hydroxylase serves as the rate-limiting enzyme in the biosynthetic pathway converting tyrosine to dopamine, playing a critical role in the neurophysiology of *Ae. aegypti*. Using quantitative PCR (qPCR), we analyzed the expression of *Aath* (the gene encoding tyrosine hydroxylase) and observed a pronounced circadian rhythm in uninfected *Ae. aegypti*. Specifically, *Aath* expression levels decreased during daylight hours and increased at night (Fig. 4A). However, in *Ae. aegypti* infected with DENV2 or ZIKV, *Aath* expression remained consistently elevated throughout the day. These findings led us to hypothesize that the circadian clock regulates *Aath* expression.

To test this hypothesis, we employed a genetic approach by injecting *dstimeless* to attenuate the expression of the *timeless* gene, effectively disrupting the biological clock in these mosquitoes (Fig. 4B). In *dstimeless*-injected mosquitoes, the circadian oscillation of *Aath* was significantly attenuated, with expression levels remaining elevated throughout the day (Fig. 4C). This disruption raised the question of whether infection with DENV2 and ZIKV broadly alters the overall circadian rhythms of mosquitoes.

To investigate further, we analyzed the circadian oscillations of key clock genes, including *tim*, *per*, and *Pdp1* at six time points (Zeitgeber time [ZT] 4, 8, 12, 16, 20, and 24) under light/dark and constant dark conditions. We found that the mRNA oscillations of *tim*, *per*, and *Pdp1* were significantly attenuated in mosquitoes infected with DENV2 and ZIKV (Fig. 4D through F).

Next, we assessed the impact of disrupted circadian rhythms on blood-feeding propensity. In mosquitoes injected with *dstimeless*, the typical peak in blood-feeding propensity observed at dusk was abolished. Instead, blood-feeding rates were consistently elevated throughout the day compared to controls (Fig. 4G). This suggests that the circadian clock plays a crucial role in regulating blood-feeding behavior and that disruption of the biological clock enhances blood-feeding efficiency.

These findings align with previous studies by Shetty et al., which reported that the knockout of circadian genes in *Ae. aegypti* increased blood-feeding propensity (28). Additionally, our results showed that the locomotor activity of *dstimeless*-injected mosquitoes was significantly higher than that of the control group injected with *dsgfp* (Fig. 4H and I), further highlighting the role of the circadian clock in mosquito behavior.

In conclusion, mosquito blood-feeding behavior is regulated by circadian rhythms. DENV2 and ZIKV disrupt the biological clock in mosquitoes, leading to increased *Aath* expression. This dysregulation enhances blood-feeding propensity and locomotor activity of mosquitoes.

### Knockdown of the *Aath* gene inhibits DENV2 transmission

**Fig 5:**
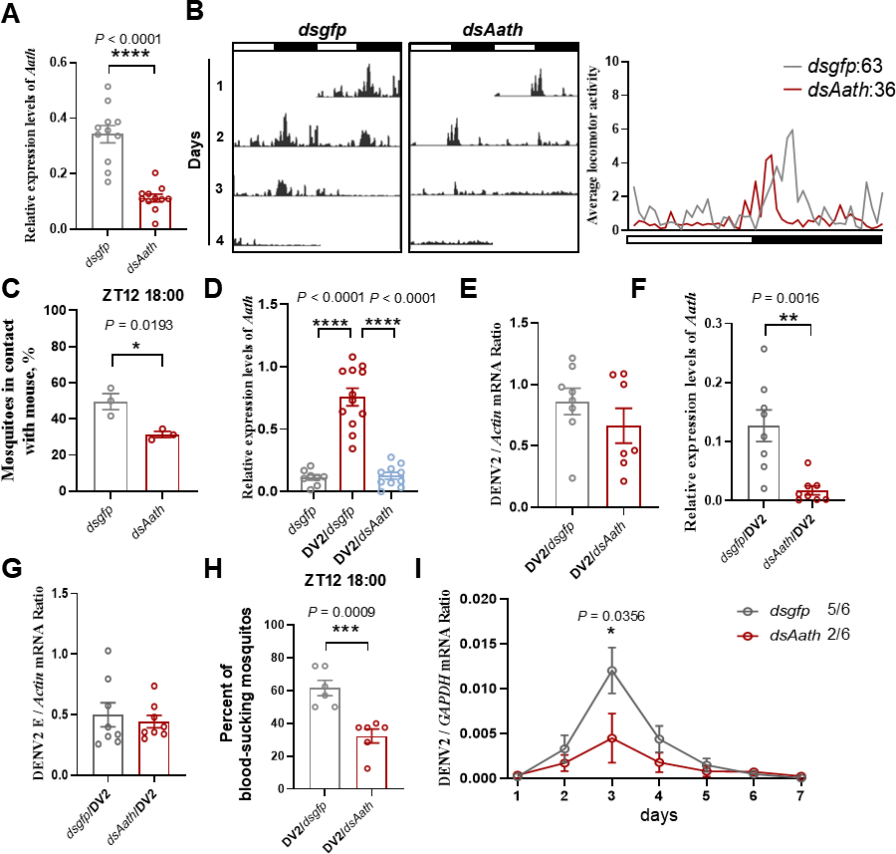
Knockdown of the *Aath* gene inhibits DENV2 transmission. (**A**) Result of RT-qPCR analysis of the relative levels of *Aath* mRNA in the head of *dsgfp*- or *dsAath*-injected mosquitoes. The *actin* gene served as an endogenous control. The error bar indicates the SEM. ****P < 0.0001 (two-sided *t*-test). (**B**) The left panel shows the locomotor activity of *dsgfp*- or *dsAath*-injected mosquitoes under LD cycles. White and black bars indicate light and dark phases, respectively. The right panel shows the average locomotor activity in the left panel. The numbers represent the total activity counts of mosquitoes over 24 h. (**C**) The blood-feeding ratio of *dsgfp*- or *dsAath*-injected mosquitoes at ZT12. The *actin* gene served as an endogenous control. The error bar indicates the SEM. *P < 0.05 (two-sided *t*-test). (**D**) Result of RT-qPCR analysis of the relative levels of *Aath* mRNA of *dsgfp*-, DENV2/*dsgfp*-, or DENV2/*dsAath*-injected mosquitoes. The *actin* gene served as an endogenous control. The error bar indicates the SEM. ****P < 0.0001 (two-sided *t*-test). (**E**) Result of the RT-qPCR analysis of the relative levels of DENV2 envelope protein in DENV2/*dsgfp*- and DENV2/*dsAath*-injected mosquitoes. The *actin* gene served as an endogenous control. The error bar indicates the SEM. (**F and G**) Result of the RT-qPCR analysis of the relative levels of *Aath* (**F**) and DENV2 envelope protein (**G**) in *dsgfp/*DENV2- and *dsAath/*DENV2-injected mosquitoes. The *actin* gene served as an endogenous control. The error bar indicates the SEM. **P < 0.01 (two-sided *t*-test). (**H**) The blood-feeding ratio of *dsgfp/*DENV2- and *dsAath/*DENV2-injected mosquitoes at ZT12. The error bar indicates the SEM. ***P < 0.001 (two-sided *t-*test). (**I**) Sera from mice bitten by control or *dsAath*-injected mosquitoes were collected daily from days 1 to 7 post-exposure. Viral RNA levels in sera were quantified via RT-qPCR. The *Mmgapdh* gene served as an endogenous control. The values 5/6 and 2/6 represent the number of mice with detectable viral RNA in sera at the peak timepoint. The error bar indicates the SEM. *P < 0.05 (two-sided *t*-test).

Our experimental results demonstrated that increased *Aath* expression induced by viral infection significantly enhances mosquito blood-feeding propensity and locomotor activity. To investigate the role of *Aath* in these processes, we knocked down *Aath* expression in mosquitoes by injecting double-stranded RNA (dsRNA) targeting *Aath* (Fig. 5A). Using the DAM2 assay, we found that locomotor activity was significantly reduced in *dsAath*-injected *Aedes aegypti* compared to mosquitoes injected with control *dsgfp* RNA (Fig. 5B). Furthermore, mosquitoes with *Aath* knockdown exhibited lower blood-feeding rates at ZT12, a time point when nearly 50% of the *dsgfp*-injected mosquitoes successfully obtained a blood meal (Fig. 5C).

To assess the impact of *Aath* knockdown on DENV2 transmission, we performed additional experiments. DENV2-infected mosquitoes were injected with either *dsAath* or *dsgfp* (Fig. 5D). Both groups accumulated similar levels of DENV genome mRNA at 7 days post-infection (Fig. 5E). In another experiment, mosquitoes were first injected with *dsAath* or *dsgfp*, followed by DENV2 infection 2 days later. After 7 days of infection, DENV levels in both groups remained comparable, indicating that *Aath* does not influence DENV2 replication in mosquitoes (Fig. 5F and G).

Despite the absence of impact on viral replication, mosquitoes with *Aath* knockdown consistently showed a lower blood-feeding ratio at ZT12 compared to mosquitoes infected with the virus alone (Fig. 5H). This suggests that *Aath* functions downstream in the molecular mechanism by which DENV2 affects mosquito blood-feeding behavior. Given the critical role of feeding behavior in mosquito-borne virus transmission, we next investigated how disruption of the dopamine pathway, via *Aath* knockdown, affects viral transmission efficiency.

To quantify transmission, AG6 mice were subjected to bites from infected mosquitoes that had been injected with either *dsAath* or *dsgfp*. As expected, the proportion of *dsAath*-injected mosquitoes that fed on mice within 10 min was markedly lower than that in the control mosquitoes (Fig. S6). Subsequently, the viral RNA levels in sera were measured. At the peak of viral RNA levels (day 3 post-exposure), viral RNA was detected in 83% (five out of six) of mice bitten by control mosquitoes, compared to 33% (two out of six) of mice that had been exposed to *dsAath*-injected mosquitoes (Fig. 5I).

Collectively, these results demonstrate that reduced *Aath* expression in mosquitoes inhibits blood-feeding behavior, which, in turn, diminishes the efficiency of DENV2 transmission.

## DISCUSSION

Mosquito-borne viruses pose significant threats to global public health. Through long-term co-evolution, a balance has been established between mosquito vectors and viruses, allowing mosquitoes to develop features that promote virus transmission (29, 30). Key behaviors of mosquito vectors, including feeding and host-seeking, can be manipulated by specific viruses to enhance transmission. For example, while DENV2 and ZIKV increase blood-feeding propensity in *Ae. aegypti* (this study), West Nile virus (WNV) reduces fecundity but elevates feeding rates in *Culex* spp. (31, 32). These contrasts underscore the virus- and vector-specific nature of behavioral modulation, cautioning against broad generalizations across flaviviruses. For instance, *Sindbis virus* (SINV)-infected mosquitoes exhibit significant changes in activation, probing, and engorgement times (33), with infected female mosquitoes requiring more time than uninfected mosquitoes to locate blood (34). Similarly, LACV infection suppresses activation and host attraction in *Aedes triseriatus* (8).

A study by Maciel-de-Freitas et al. demonstrated that dengue virus-infected *Aedes aegypti* are more likely to re-feed compared to uninfected mosquitoes, suggesting that virus infection can increase vectorial capacity by modifying blood-feeding behavior. While WNV infection reduces mosquito fecundity, it concurrently increases blood-feeding rates (31, 32). Tallon et al. showed that DENV1 affects locomotion and odor-mediated behavior in *A. aegypti*. Specifically, mosquitoes 4–6 days post-infection displayed increased locomotion without altering odor-driven host-seeking responses, whereas females 14–16 days post-infection became less active but more sensitive to human odors, reflecting the progression of infection within the mosquito (6). Similarly, recent work by Pompon’s group (7) and independent studies on SINV (33) and WNV (32) demonstrate that diverse arboviruses can alter mosquito feeding behavior through distinct mechanisms. For instance, DENV2-induced behavioral changes may triple transmission efficiency in controlled settings (7), whereas WNV indirectly enhances feeding rates via fitness trade-offs (32). This diversity highlights the need for pathogen-specific mechanistic studies. In contrast, studies on DENV3 and DENV2 provide mixed results, while Platt et al. observed longer feeding periods in DENV3-infected mosquitoes (35). Putnam and Scott reported no significant changes in feeding duration or efficiency for DENV2-infected mosquitoes (36). The inconsistencies could be attributed to potential methodological biases or technical artifacts. Interestingly, plant viruses can also modulate vector behavior. For example, barley yellow striate mosaic virus directly enhances the locomotor activity of small brown planthoppers to facilitate transmission (37).

In our study, using the long-established Rockefeller strain of *Ae. aegypti*, we observed that DENV2- and ZIKV-infected *Ae. aegypti* spp. exhibit increased blood-feeding propensity and locomotor activity. It is important to note that this strain has been reared in the laboratory for over a century, and its behavior may have diverged from wild populations due to the artificial rearing conditions, such as simplified light cycles and absence of ecological stressors. While uninfected *Ae. aegypti* displayed peak blood-feeding activity at dusk, virus-infected mosquitoes maintained elevated feeding propensity throughout the day (Fig. 1B). This observation suggests that DENV2 and ZIKV can manipulate mosquito feeding behavior to enhance transmission potential, but further validation in field-caught mosquitoes or semi-field systems is needed to confirm the relevance of these findings in natural settings. The lighting conditions in the laboratory, often with simple on-off cycles set for convenience, likely differ from natural crepuscular lighting, which could impact the mosquitoes’ behavior. Therefore, the results obtained in this study may not directly reflect the situation in the real world.

Beyond viruses, other pathogens, such as *Plasmodium*, also alter mosquito behavior (38). *Plasmodium*-infected *Anopheles gambiae* spp. are more attracted to human odors (39), and *Plasmodium gallinaceum*-infected *Ae. aegypti* spp. show heightened olfactory responses to guinea pig odors (40). In *Anopheles stephensi* infected with *P. yoelii*, changes in host attraction correlate with altered responsiveness of odorant receptors, depending on the stage of infection (41). These studies suggest that both arboviruses and *Plasmodium* parasites may share neurophysiological mechanisms to modulate vector behavior. A critical question remains: are these behavioral modifications induced by arboviruses distinct from or analogous to those triggered by *Plasmodium*?

Determining whether pathogen factors directly affect the mosquito brain or act indirectly through host-mediated mechanisms remains challenging. For example, some parasites do not alter host behavior until reaching their late stages in the definitive host (42). In our study, we used thoracic microinjection to bypass the midgut barrier and ensure robust viral replication in mosquito tissues. Assays conducted 7 days post-injection confirmed that DENV2 and ZIKV had reached late-stage infection, fully infecting neural tissues. At this stage, the tyrosine-dopamine pathway was induced. However, we could not ascertain whether this activation was a general symptom of infection or a specific function of viral factors. Future studies could explore virus-induced effects during earlier infection stages.

Neurotransmitters, such as dopamine, play a pivotal role in resculpting neural circuits and modifying insect behavior. Dopamine has been implicated in locomotion, pupation, and eclosion in mosquitoes and *Drosophila* (43, 44). Reduced expression of dopamine receptors significantly decreases locomotor activity in flies (45), while receptor agonists enhance activity in honeybees (46). In our study, we found that the dopamine pathway was closely associated with increased locomotor activity in virus-infected mosquitoes. Additionally, other neurotransmitters, such as 5-HT, known to regulate feeding processes and salivary secretion (47–49), may also be influenced by viral infection. Notably, DENV2 alters the expression of structural, secreted, and metabolic proteins in mosquito salivary glands (50), potentially linking viral effects on neurotransmitters to feeding behavior.

Circadian rhythms govern many mosquito behaviors, including blood feeding (51). While *Anopheles gambiae* blood feeding is regulated by circadian rhythms (52), our findings reveal that DENV2 and ZIKV infections suppress these rhythms in *Ae. aegypti*, disrupting peak blood-feeding propensity at dusk. We also observed that the *Aath* gene, central to the tyrosine-dopamine pathway, is influenced by circadian rhythms. It is plausible that virus-induced disruptions extend to other rhythmic genes related to mosquito behavior, such as chemosensory and odorant receptor genes. Whether peripheral biological clocks in mosquitoes are similarly affected remains unknown and warrants further investigation.

This study highlights several critical limitations. First and foremost, all experiments were conducted under controlled laboratory conditions using long-term adapted mosquito strains (e.g., Rockefeller *Ae. aegypti*), which may exhibit behavioral divergence from wild populations due to artificial rearing environments. Next, while our findings provide a mechanistic framework for DENV2- and ZIKV-induced behavioral modulation, direct extrapolation to natural settings requires validation in field-caught mosquitoes or semi-field systems. Notably, field studies report crepuscular blood-feeding peaks in *Ae. aegypti*, aligning with our observations in uninfected laboratory mosquitoes (Fig. 1B). This consistency suggests that laboratory models can capture baseline behavioral rhythms, but virus-induced disruptions observed here may be attenuated or amplified in ecologically complex settings. Additionally, our mosquito biting behavior assays were conducted using AG6 mice rather than humans, raising questions about the generalizability of increased feeding behavior to human hosts. Lastly, our focus on DENV2 and ZIKV precludes generalization to other flaviviruses, particularly those transmitted by behaviorally divergent vectors like *Culex* spp. Future studies should compare viral effects across laboratory and field strains, incorporate twilight-mimicking light cycles, and expand to understudied flaviviruses to delineate conserved versus virus-specific mechanisms.

## MATERIALS AND METHODS

### Untargeted metabolomics analysis

The heads of mosquitoes (20 per sample) were individually ground with liquid nitrogen, and the homogenate was resuspended with prechilled 80% methanol by well vortex. Samples were incubated on ice for 5 min and centrifuged at 15,000 × *g* at 4°C for 20 min. A portion of the supernatant was diluted with liquid chromatography-mass spectrometry (LC-MS) grade water to a final concentration of 53% methanol. The diluted samples were transferred to fresh Eppendorf tubes and centrifuged again at 15,000 × *g* at 4°C for 20 min. Finally, the supernatant was injected into the liquid chromatography-tandem mass spectrometry (LC-MS/MS) system analysis. Data processing and analyses were performed by Novogene (Beijing, China).

### Viruses, mice, and mosquitoes

*Aedes aegypti* (Rockefeller strain), *Aedes albopictus* (China Jiangsu strain), and *Aedes albopictus* (China Ruili strain) mosquitoes were provided by Beijing Institute of Microbiology and Epidemiology, reared under standard conditions (28°C, 80% relative humidity), and provided with a sugar solution.

C57BL/6 mice deficient in type I and II interferon receptors (AG6 mice) were donated by the Institute Pasteur of Shanghai, Chinese Academy of Sciences. Mice were bred and maintained at the Tsinghua University animal facility in a pathogen-free environment. Six- to eight-week-old female mice were used for all experiments.

DENV2 virus (43 strains, AF204178) and ZIKV virus (PRVABC59 strain, KU501215) were propagated in C6/36 cells maintained in Dulbecco’s modified Eagle medium at 37°C with 5% CO_2_. Viral titers were determined using plaque assays. For mosquito infections, 300 nL of virus stock (>1 × 10⁵ CFU) was microinjected into the thorax of 1-week-old mosquitoes, followed by a 7 day incubation to allow viral replication.

### HPLC analysis

Ten mosquito heads were homogenized in 100 µL of prechilled 80% methanol (HPLC-grade) in a 1.5 mL Eppendorf tube. The homogenates were ground for 2 min with a tissue grinder on dry ice, vortexed for 1 min at 4°C–8°C, and incubated at −80°C for 2 h or overnight. Samples were centrifuged at 14,000 × *g* at 4°C for 20 min, and the supernatant was transferred to a new tube. The pellet was dried using N_2_ gas at room temperature, and the samples were analyzed immediately. For dopamine detection, the samples were dissolved in 100 µL of mobile phase, and 20 µL was injected into the HPLC.

### Locomotor activity analysis

Mosquito locomotor activity was assessed using the DAM2 system (TriKinetics, Waltham, MA). Mosquitoes were individually placed into glass tubes with one end containing cotton soaked in 10% sucrose. Tubes were placed in a chamber maintained at 28°C, 80% humidity, and a 12 h light/dark cycle. Movement across an infrared beam in the middle of the tube was recorded every 30 min using DAM data collection software. At least two replicates were conducted, and data were analyzed using ActogramJ to generate actograms.

### Mosquito blood-sucking ratio test

Groups of 20–30 mosquitoes were placed in a 20 × 20 × 20 cm net cage. After immobilization by chilling, the mosquitoes were allowed to recover. A fixed AG6 mouse was placed on top of the cage, providing a blood source for 30 min. Afterward, the mouse was removed, and the number of blood-fed mosquitoes was counted to calculate the blood-sucking ratio.

### Reverse transcription quantitative PCR

Total RNA was extracted from homogenized mosquito corpses or heads using RNeasy Mini Kit (74106, Qiagen). Complementary DNA (cDNA) was synthesized using the iScript cDNA synthesis kit (Bio-Rad, Cat. 170-8890). Quantitative PCR of the *Aedes aegypti* gene and virus genomes was carried out on the Bio-Rad CFX96 Real-Time PCR System using SYBR qPCR amplifications. The primers used for these analyses are shown in Table S2. *Aedes aegypti actin* and *RPS17* gene served as an endogenous control.

### Immunohistochemistry

Dissected mosquito heads were fixed in the 4% paraformaldehyde with 0.1% Triton X-100 at 4°C overnight. Samples were washed three times with PBS and blocked with 5% bovine serum albumin (BSA) at 37°C for 1 h. They were incubated with 4G2 antibodies (1:200 dilution) at 37°C for 1 h, followed by three washes in PBS containing 0.1% BSA. Secondary antibody (anti-rabbit IgG conjugated with Alexa Fluor 488, 1:500 dilution) was added and incubated at 37°C for 30 min. After washing, samples were imaged using a Ziss LSM 780 confocal microscope. Negative controls were incubated with Alexa Fluor 488-conjugated IgG alone.

### Gene silencing and metabolite microinjection in mosquitoes

Fragments of *Aath* and *Aatimeless* genes were amplified using primers containing T7 RNA polymerase promoter sequences (Table S2). The purified PCR products were used as templates for dsRNA synthesis using the T7 High Efficiency Transcription Kit (Trangen). *dsgfp* was used as a negative control. Female mosquitoes were microinjected with 1 µg/300 nL of dsRNA into the thorax under anesthesia on a cold tray. To investigate the effect of different metabolites on mosquito blood-sucking behavior, the compounds were dissolved in sterile PBS at a concentration of 100 mM and microinjected into the thorax of mosquitoes. The compounds used in this study were obtained from Macklin (GABA, CAS: 57659-38-8; citric acid, CAS: 77-92-9; serotonin, CAS: 56-69-9; syringic acid, CAS: 530-57-4; NAD, CAS: 53-84-9; L-carnitine, CAS: 541-15-1; D-panthenol, CAS: 81-13-0; N-acetyl-L-tyrosine, CAS: 537-55-3).

### Mosquito-borne viral infection

Six-week-old female AG6 mice were exposed to 10 dsRNA-treated, virus-infected mosquitoes for 10 min. Blood samples were collected from the tail daily for 7 days to measure viremia.

### Data analysis

Statistical analyses were performed using GraphPad Prism version 8 statistical software. Data with normal distributions were analyzed using two-tailed, paired *t*-tests. Blood-feeding ratios and reverse transcription quantitative PCR results in Fig. 1B and 4A through C were analyzed using two-way analysis of variance followed by Tukey’s post hoc test. Asterisks indicate statistical significance (*P < 0.05, **P < 0.01, ***P < 0.001, ****P < 0.0001).

## Data Availability

All data generated or analyzed during this study are included in this article and its supplemental materials. The metabolomics data reported in this paper have been deposited in OMIX, China National Center for Bioinformation/Beijing Institute of Genomics, Chinese Academy of Sciences (accession no. OMIX009397). The NCBI accession numbers of the described genes in this study are as follows: *Aatimeless* (XM_021847394), *Aath* (XM_011495191), *Aaperiod* (XM_021854835), *Aaactin* (XM_001649482), *Aapdp1* (XM_021841174), *AaRPS17* (XM_001648517), and *MmGAPDH* (NM_001411844.1).

## SUPPLEMENTAL MATERIAL

Data S1 mbio.03968-24-s0001.xlsx Metabolism in heads of WT and DENV2- and ZIKV-infected mosquitoes.

Supplemental figures and tables mbio.03968-24-s0002.docx Fig. S1 to S6; Tables S1 and S2.

ASM does not own the copyrights to Supplemental Material that may be linked to, or accessed through, an article. The authors have granted ASM a non-exclusive, world-wide license to publish the Supplemental Material files. Please contact the corresponding author directly for reuse.
