# Supplementary material for: DENV2 and ZIKV modulate the feeding behavior of Aedes aegypti by altering the tyrosine-dopamine pathway
Source: mBio. 2025 Apr 29;16(6):e03968-24. doi: 10.1128/mbio.03968-24 (PMC12153320; doi:10.1128/mbio.03968-24)
Supplement: Supplemental figures and tables — Fig. S1 to S6; Tables S1 and S2. [file mbio.03968-24-s0002.docx]

**DENV2 and ZIKV modulate the feeding behavior of *Aedes aegypti* by altering tyrosine-dopamine pathway**

Dongmin Gao^1^, Ruixu Jiang^1^, Zhaoyang Wang^1,2,3^, Jichen Niu^1^, Gang Wang^1^, Yicheng Wang^1^, Yan Liang^1^, Yibin Zhu^1,2,3^, Gong Cheng^1,2,3*^

Gong Cheng

gongcheng@mail.tsinghua.edu.cn

**This PDF file includes:**

Supporting text

Figures S1 to S6

Tables S1 to S2

Legends for Datasets S1

**Fig. S1.** Pearson correlation coefficients between QC samples, left panel is metabolite data identified by the positive ion model (POS), right panel is metabolite data identified by the negative ion model (NEG).


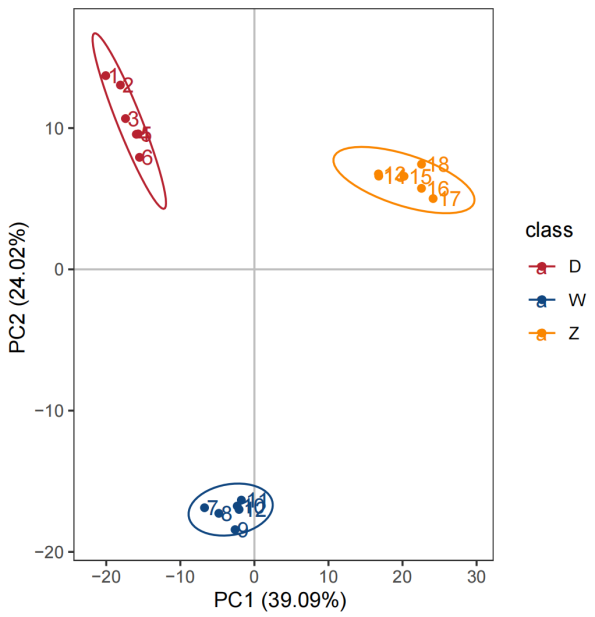
**Fig. S2.** Principal component analysis of total samples, left panel is the sample of positive ion model, right panel is the sample of negative ion model.

Fig. S3. KEGG functional and taxonomic annotation of the identified metabolites, left panel is the metabolites in positive ion model, right panel is the metabolites in negative ion model.

**Fig. S4.** N-ace-L-tyrosine levels of healthy (W), DENV2-infected (D) and ZIKV-infected (Z) A. aegypti. ***P < 0.001, ****P < 0.0001 (two-sided t test).

Fig.S5. Dopamine levels in the heads of L-DOPA-injected mosquitoes by HPLC, PBS-injected as control. ***P < 0.001 (two-sided t test).


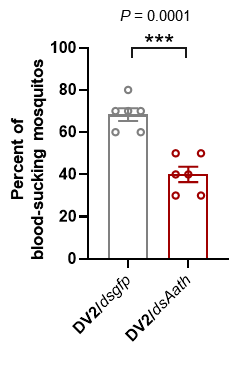
Fig.S6. The blood-feeding ratio of 10 DENV2/*dsgfp*- or DENV2/*dsAath*-injected mosquitoes after biting AG6 mice within 10 min. ***P < 0.001 (two-sided t test).

Table S1. The list of metabolisms induced and decreased by DENV2 and ZIKV

Table S2. The list of primers used in the study.

Data S1. (separate file) The metabolisms in head of WT, DENV2- and ZIKV- infected mosquitoes
